# Chemoradiotherapy (Gemox Plus Helical Tomotherapy) for Unresectable Locally Advanced Pancreatic Cancer: A Phase II Study

**DOI:** 10.3390/cancers11050663

**Published:** 2019-05-13

**Authors:** Alessandro Passardi, Emanuela Scarpi, Elisa Neri, Elisabetta Parisi, Giulia Ghigi, Giorgio Ercolani, Andrea Gardini, Giuliano La Barba, Flavia Pagan, Andrea Casadei-Gardini, Martina Valgiusti, Fabio Ferroni, Giovanni Luca Frassineti, Antonino Romeo

**Affiliations:** 1 Department of Medical Oncology, Istituto Scientifico Romagnolo per lo Studio e la Cura dei Tumori (IRST) IRCCS, Via P. Maroncelli n. 40, 47014 Meldola, Italy; alessandro.passardi@irst.emr.it (A.P.); casadeigardini@gmail.com (A.C.G.); martina.valgiusti@irst.emr.it (M.V.); luca.frassineti@irst.emr.it (G.L.F.); 2 Unit of Biostatistics and Clinical Trials, IRST-IRCCS, Via P. Maroncelli n. 40, 47014 Meldola, Italy; flavia.pagan@irst.emr.it; 3 Radiotherapy Unit, IRST-IRCCS, Via P. Maroncelli n. 40, 47014 Meldola, Italy; elisa.neri@irst.emr.it (E.N.); elisabetta.parisi@irst.emt.it (E.P.); giulia.ghigi@irst.emr.it (G.G.); antonino.romeo@irst.emt.it (A.R.); 4 General and Oncologic Surgery Unit, Morgagni-Pierantoni Hospital, AUSL Romagna, Via C. Forlanini n. 34, 47121 Forlì Italy; giorgio.ercolani@auslromagna.it (G.E.); andrea.gardini@auslromagna.it (A.G.); giuliano.labarba@tin.it (G.L.B.); 5 Department of Medical and Surgical Sciences, University of Bologna, Via Massarenti n. 9, 40138 Bologna, Italy; 6 Radiology Unit, IRST IRCCS, Via P. Maroncelli n. 40, 47014 Meldola, Italy; fabio.ferroni@irst.emr.it

**Keywords:** pancreatic cancer, neoadjuvant therapy, GEMOX, radiotherapy

## Abstract

The aim of the study was to evaluate the safety and efficacy of a new chemo-radiotherapy regimen for patients with locally advanced pancreatic cancer (LAPC). Patients were treated as follows: gemcitabine 1000 mg/m^2^ on day 1, and oxaliplatin 100 mg/m^2^ on day 2, every two weeks (GEMOX regimen) for 4 cycles, 15 days off, hypofractionated radiotherapy (35 Gy in 7 fractions in 9 consecutive days), 15 days off, 4 additional cycles of GEMOX, restaging. From April 2011 to August 2016, a total of 42 patients with non resectable LAPC were enrolled. Median age was 67 years (range 41–75). Radiotherapy was well tolerated and the most frequently encountered adverse events were mild to moderate nausea and vomiting, abdominal pain and fatigue. In total, 9 patients underwent surgical laparotomy (5 radical pancreatic resection 1 thermoablation and 3 explorative laparotomy), 1 patient became operable but refused surgery. The overall resectability rate was 25%, while the R0 resection rate was 12.5%. At a median follow-up of 50 months, the median progression-free survival and overall survival were 9.3 (95% CI 6.2–14.9) and 15.8 (95% CI 8.2–23.4) months, respectively. The results demonstrate the feasibility of a new chemo-radiotherapy regimen as a potential treatment for unresectable LAPC.

## 1. Introduction

Pancreatic cancer has an expected incidence of 56,770 new cases and 45,750 deaths in 2019 in the United States alone. It ranks as the 7th leading cause of cancer related mortality and has a fatal prognosis with 5-year survival rate of about 9% [1,2]. At the time of diagnosis, about 30% of patients have locally advanced unresectable disease [1], i.e., with radiological evidence of celiac axis or superior mesenteric artery encasement of more than 180, superior mesenteric or portal vein distortion not allowing for safe resection and replacement, and aortic or nodal involvement beyond the field of resection (stage T4) [3]. The prognosis of patients with locally advanced pancreatic cancer (LAPC) is poor, with a median overall survival (OS) of 9 to 11 months [1]. In this setting chemotherapy (CT) alone or chemoradiotherapy (CTRT) have been generally accepted as standard therapeutic approaches, although the role of CTRT remains controversial.

Since the early 1980s, fluorouracil-based CTRT was shown to be more efficacious than radiotherapy alone [4]. Later, gemcitabine was adopted as the standard of care, replacing CTRT also in patients with LAPC, since results from randomized trials comparing CTRT with CT alone had been contradictory [5,6,7]. Recently, with more active CT regimens available, such as FOLFIRINOX [8] and gemcitabine/albumin-bound paclitaxel, the indication to CTRT has been further reduced.

Some retrospective studies have suggested that induction CT administered before concurrent CTRT could improve survival [9,10]. Such a therapeutic strategy may allow to select patients without early progression of disease as those more likely to benefit from subsequent CTRT. Unfortunately, the LAP07 Randomized Clinical Trial did not show any clear survival benefit with the addition of conventional CTRT following gemcitabine monotherapy [11]. In fact the median OS was not significantly different between CT (16.5 months, 95% confidence interval [CI], 14.5–18.5 months) and CTRT (15.2 months, 95% CI, 13.9–17.3 months; hazard ratio [HR], 1.03; 95% CI, 0.79–1.34; *p*  = 0.83). However, the study did demonstrate significantly decreased local progression (32% vs. 46%, *p*  =  0.03) with minimal increase in treatment-related toxicity in the CTRT arm.

Interest in hypofractionated radiotherapy has increased alongside with the recognition of a potential improvement in therapeutic efficacy with treatment delivered in larger-sized fractions. This strategy might permit to deliver higher doses of radiation to the tumor whilst reducing patients’ time off full dose chemotherapy, as most regimens use 5 fractions or less. However, sparing of normal tissues is essential for a good therapeutic outcome. Preliminary results from non-randomized trials of stereotactic body radiotherapy (SBRT) have been recently published, demonstrating good local control (approximately 80% at 1 year) but still poor survival rates with most patients dying from metastatic diseases [12,13,14,15,16,17,18,19].

We carried out a pilot study to assess the feasibility and efficacy of a CTRT split-course regimen with gemcitabine and oxaliplatin (GEMOX) plus Helical Tomotherapy in patients with unresectable LAPC. From November 2004 to July 2008, 30 patients were enrolled and received GEMOX for 3–4 cycles, followed by radiotherapy (25 Gy, 5 fractions) and further 3–4 cycles of GEMOX. Potentially resectable patients were submitted to surgery, while unresectable responders received further GEMOX and radiotherapy (3 fractions). After CTRT, 14 patients obtained a partial tumor response (44%), among them 8 patients (24%) underwent surgical laparotomy (7 radical pancreatic resection and 1 explorative laparotomy) [20]. The median PFS and OS were 11 months (95% CI 8–13) and 14 months (95% CI 12–18), respectively. 1- and 2-year overall survival rates were 63% (95% CI 46–81) and 21% (95% CI 4–39), respectively.

The purpose of this study was to assess whether continuous hypofractionated (7 fractions) radiotherapy in association with the same CT regimen was safe and whether it could further increase resectability rate in unresectable LAPC.

## 2. Results

### 2.1. Patient Characteristics

From April 2011 to August 2016, 42 patients with non-resectable LAPC were prospectively enrolled onto the trial, 2 of whom were considered not evaluable for eligibility criteria violation. Patient characteristics are listed in Table 1.

The median age at the time of diagnosis was 67 years (range 41–75). 62.5% of patients were female, ECOG Performance status was 0 in 25 patients (62.5%) and 1–2 in 15 (37.5%). 11 (27.5%) and 29 (72.5%) patients had stage II and III disease, respectively.

### 2.2. Treatment Administration

Patients received a total of 233 cycles of CT, with a median of 6 cycles per patient (range 1 to 8 cycles). Twenty-eight patients (70%) regularly completed CTRT according to study protocol. Early interruption of treatment was reported for the remaining 12 patients (8 for early progression, 4 for patient or investigator’s decision). In particular radiotherapy was not administered in 10 patients, and was administered at lower doses, as a palliative treatment, to 2 patients.

### 2.3. Toxicity

A safety evaluation was performed after the first 11 patients were enrolled in Step A. Among them 1 case of toxicity requiring the radiotherapy treatment discontinuation was observed (grade 4 abdominal pain). As provided, 7 additional patients were recruited, who did not show significant toxicity. For this reason, the study continued in step B.

All 40 patients were evaluable for toxicity. Treatment was generally well tolerated, and the most common adverse events are listed in Table 2. 

Overall, 6 cases of grade 4 toxicity were recorded: 2 neutropenia, 1 febrile neutropenia, 1 thrombocytopenia without bleeding, 1 diarrhoea and 1 abdominal pain. Haematological toxicity was as expected. We observed 5 cases of grade 3–4 neutropenia (12.5%), 2 cases of grade 3 anaemia (5%), 6 cases of grade 3–4 thrombocytopenia. The most frequently encountered non haematological adverse events were mild to moderate nausea and vomiting, diarrhoea or constipation and fatigue. Only 7 patients (17.5%) experienced grade 2–3 and fully reversible peripheral neuropathy.

Overall, except for the above-mentioned case of grade 4 abdominal pain, adverse reactions due to radiotherapy were tolerable and fully reversible. Moreover, no late toxicities such as gastrointestinal ulcer or biliary or duodenal obstruction were reported.

### 2.4. Efficacy

Overall, 9 patients underwent surgical laparotomy (5 radical pancreatic resection 1 thermoablation and 3 explorative laparotomy), 1 patient became operable but refused surgery. The overall resectability rate was 25% (95% CI 11.6–38.4), while the R0 resection rate was 12.5% (95% CI 2.25–22.75). Clinico-pathological characteristics of resected patients are shown in Table 3. 

None of the radically operated patients received any adjuvant treatment. Among the 5 patients who underwent R0 resection 2 patients are still alive without signs of recurrence after 28 and 81 months, while one of them died from surgical complications and another 2 of them died from distant metastases. The patient treated with thermoablation after CTRT is still alive and progression-free after 65 months.

All 40 patients were evaluable for response, among them 5 patients obtained a partial tumor response, for an ORR of 12.5% (95% CI 2.25–22.75). 20 patients showed stable disease, while 15 progressed. Seventeen patients received second line chemotherapy (12 FOLFIRI, 5 gemcitabine/albumin-bound paclitaxel).

**Figure 1:**
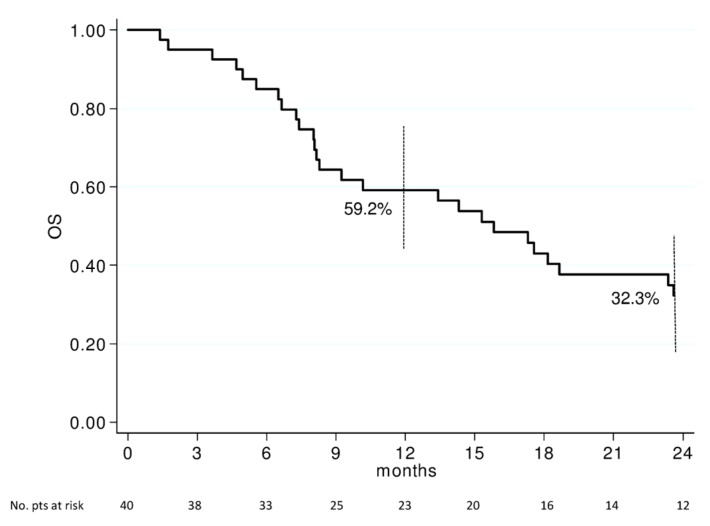
Overall survival of 40 patients with non-resectable locally advanced pancreatic cancer (LAPC). Vertical bars represent 95% Confidence Interval of survival probability at 1 and 2 years. OS: Overall Survival; pts: patients.

**Figure 2:**
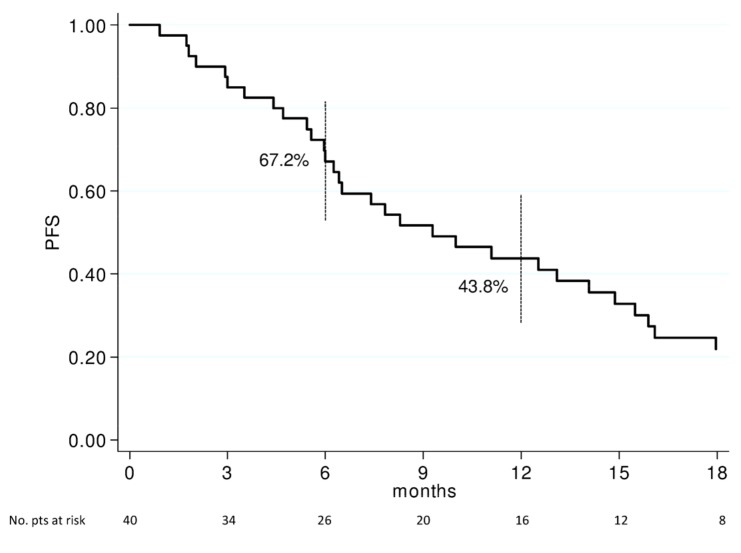
Progression-free survival of 40 patients with non-resectable LAPC. Vertical bars represent 95% Confidence Interval of survival probability at 6 and 12 months. PFS: Progression-Free Survival; pts: patients.

After a median follow-up time of 50 months, the median PFS and OS were 9.3 (95% CI 6.2–14.9) and 15.8 (95% CI 8.2–23.4) months, respectively. 1- and 2-year OS rates were 59.2% (95% CI 43.8–74.6) and 32.3% (95% CI 18.4–47.2), respectively (Figure 1). 6- and 12-months PFS rates were 67.5% (95% CI 52.5–81.8) and 43.8% (95% CI 28.1–59.4), respectively (Figure 2).

## 3. Discussion

Although CTRT have played a pivotal role in the treatment of LAPC, the optimal treatment strategy is still a matter of debate, and CT with gemcitabine can be still considered the recommended standard [21]. Moreover, published data indicate that only a minority of patients with LAPC become radically resectable after neoadjuvant treatment, and that median OS is still about 9–11 months.

In this context, the role of SBRT has been analyzed in some prospective trials. The advantages of administering SBRT are related to the shorter treatment duration which permits a better integration with CT. In fact, while conventionally fractionated radiotherapy requires at least 5 or 6 weeks of treatment in combination with suboptimal CT, an hypofractionated treatment allows fewer interruptions in full dose CT and may improve treatment outcomes. Early phase 1/2 trials with 25 Gray single-fraction SBRT demonstrated some activity but high rates of late grade 2 to 4 toxicity [12,22,23,24,25]. In particular, given the location of the pancreas (next to the stomach, small bowel and biliary structures), the most commonly reported subacute and late toxicities included gastrointestinal ulcer, with perforation and/or bleeding, and biliary or duodenal obstruction due to fibrosis. Later trials with fractionated SBRT, i.e., with smaller doses per fraction, showed a more favorable toxicity profile and reduced risk of complications, while maintaining a good local control.

About local and regional recurrence after SBRT for pancreatic adenocarcinoma, in-field and out of field events rate remains largely unknown. Nevertheless, in SBRT trials, elective regional nodes are deliberately excluded from the target volume in order to minimize toxicity. The target volume is indeed usually restricted to the gross tumor volume plus an anisotropic margin only. Due to the curative intent of our trial, the elective nodal irradiation (ENI) was included in the treatment planning although no consensus exists about this item. Despite this choice in our case series the toxicity profile was favorable, as acute toxicity was minimal and severe late toxicities were not reported.

The present trial met its primary endpoint (resectability rate 20%), moreover median PFS (9.3 months) and OS (15.8 months) are noteworthy, with 59% and 32% of treated patients alive at 1 and 2 years respectively. However, we reported a relatively low ORR and resection rate (12.5%), which is consistent with observations from other studies, in particular no radiological complete responses were seen.

It bears mentioning that 2 retrospective studies in borderline resectable pancreatic cancer have suggested that radiographic response and pathologic response after neoadjuvant treatment do not correlate [26,27]. For this reason any decision about the diagnostic management and tumor resectability should involve multidisciplinary consultation at high-volume centers, moreover an explorative laparotomy should be considered for all patients with local control and no systemic spread of disease. A retrospective evaluation was recently published suggesting a role of CA19.9 response in the selection of LAPC patients more likely to benefit from surgery after neoadjuvant treatment [28].

## 4. Materials and Methods 

### 4.1. Patient Eligibility

Inclusion criteria were: histologically or cytologically confirmed diagnosis of pancreatic cancer; Stage IIA, IIB or III disease (according to American Joint Committee on Cancer-AJCC TNM 6th edition, 2002); inoperable disease (by radiological and surgical evaluation); age ≥18 years and ≤75 years; life expectancy 12 weeks or more; Eastern Cooperative Oncology Group-ECOG performance status 0–2; normal organ and marrow function (leukocytes ≥3000/μL, absolute neutrophil count ≥1500/μL, platelets ≥100,000/μL, total bilirubin ≤1.5 × upper limit of normality-ULN, aspartate transferase-AST (serum glutamic oxaloacetic transaminase-SGOT)/alanine transferase-ALT (serum glutamate-pyruvate transaminase-SGPT) ≤2.5 × ULN, Creatinine ≤1.5 × ULN). 

Exclusion criteria were: prior chemotherapy or radiotherapy; stage IV disease; participation in another clinical trial with any investigational agents within 30 days prior to study screening; previous malignancy except cervical carcinoma in situ, adequately treated basal cell carcinoma, superficial bladder tumors or other malignancies curatively treated >5 years before study entry; history of allergic reactions attributed to compounds having chemical or biologic composition similar to gemcitabine and oxaliplatin or other agents used in the study; active brain or leptomeningeal disease; uncontrolled intercurrent illness including, but not limited to, ongoing or active infection, symptomatic congestive heart failure, unstable angina pectoris, cardiac arrhythmia, or psychiatric illness/social situations that would limit compliance with study requirements.

### 4.2. Treatment Plan

**Figure 3:**
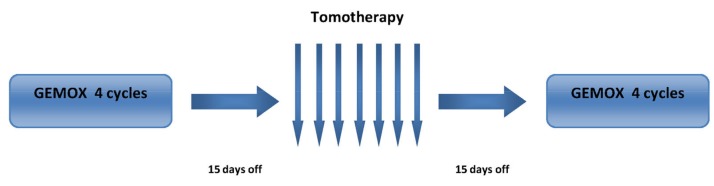
Treatment strategy. GEMOX: Gemcitabine (GEM) 1000 mg/m^2^ on day 1, Oxaliplatin (OX) 100 mg/m^2^ on day 2, every two weeks. Tomotherapy: 35 Gy in 7 fractions in 9 consecutive days, one session per day excluding Saturday and Sunday.

Patients were treated as follows (Figure 3): Gemcitabine (GEM) 1000 mg/m^2^ on day 1, and Oxaliplatin (OX) 100 mg/m^2^ on day 2, every two weeks (GEMOX regimen) for 4 cycles, 15 days off, hypofractionated radiotherapy (35 Gy in 7 fractions in 9 consecutive days, one session per day excluding Saturday and Sunday), 15 days off, 4 additional cycles of GEMOX. 

Patients then underwent restaging and were evaluated for surgery. Potentially resectable patients underwent surgery, while unresectable responders received further cycles of GEMOX or GEM alone as maintenance, at the discretion of the investigator.

Radiotherapy was performed using helical tomotherapy. Patients were initially scanned on a contrast enhanced computed tomography simulator using 3-mm slice thickness to define the treatment plan according to tumor mass, lymph nodes and organs at risk. Like other IMRT techniques, inverse planning for tomotherapy required comprehensive contouring of organs at risk as well as the identification of the regions to be treated: the gross tumor volume (GTV), including the tumor mass; the clinical tumor volume (CTV) 1, containing lymph nodal metastases; and the CTV 2 that refers to regional lymph nodes (at risk of microscopic diffusion). Before each treatment fraction, patients underwent daily scanning and were repositioned after co registration of the images with the simulation computed tomography scan. Liver, kidneys, small bowel, stomach and bone marrow were found to be organs at risk. Treatment was delivered by helical tomotherapy at a dose of 35 Gy (with an inhomogeneous dose distribution inside the target volume of up to 30% of the prescription dose) in 7 daily fractions over 9 days on the GTV; 28 Gy-35 Gy was administered on the CTV1-CTV2 on the basis of nodal status.

No adjuvant treatment was considered for patients who underwent resection.

### 4.3. Statistical Considerations

This was an open-label, single-arm, single-institutional, phase II study to evaluate the safety and the proportion of resectable patients with LAPC treated with an innovative CTRT scheme. The study consisted of two steps: step A to identify the safety of the radiotherapy treatment and step B to identify the proportion of resectable patients at the end of the treatment.

The sample size for Step A was calculated assuming that the probability to report a toxicity involving radiotherapy treatment discontinuation with the new treatment is less than 20%. 11 patients were to be evaluated for toxicity: if no toxicity involving the radiotherapy treatment discontinuation was observed in 11 patients, the treatment could be considered safe with a probability >90%; if 1 case of toxicity was observed, 7 more patients would be evaluated. If 2 or more cases of toxicity involving the radiotherapy treatment discontinuation were observed, the study would be stopped and another kind of radiotherapy schedule would be designed. If the radiotherapy treatment was considered safe, the study would continue in Step B and the patients enrolled in the first step would be also evaluated in this second step.

The sample size of Step B was calculated considering the hypothesis to increase the proportion of resectable patients by at least 15% with the new CTRT treatment. Considering P0 as the expected proportion of resectable patients and *P*1 as the proportion of resectable patients with the new CTRT treatment, 40 patients were enough to show an increase of the proportion of resectable patients from *P*0 = 10% to *P*1 = 25% (alpha = 0.1, one-side test, and power of 90%). The treatment could be considered active if at least 7 patients out of the 40 patients enrolled would be resectable.

Efficacy and toxicity analyses were performed on all patients who received at least one dose of study treatment. Resectability was defined as the absence of: superior mesenteric artery and celiac trunk encasement, invasion of aorta or inferior vena cava, occlusion of mesenteric or portal vein, distant metastases. Objective tumor response was assessed using Response Evaluation Criteria in Solid Tumors (RECIST) criteria. The objective tumor response rate (ORR) was defined as the proportion of the intention-to-treat (ITT) population showing a complete or partial response, if confirmed ≥4 weeks later. OS was counted from the date of registration to the date of death due to any cause or last date the patient was known to be alive (censored observation). Progression-Free Survival (PFS) was counted from the date of registration to the date of the first observation of documentation of objective disease progression/local disease progression or death due to any cause, whichever occurred first, or last tumor evaluation. Descriptive statistics were reported as proportions, median values and ranges. Kaplan-Meier estimates were used in the analysis of time-to-event variable and the 95% confidence interval (95% CI) was computed using the Greenwood method. Statistical analyses were carried out with SAS Statistical software (version 9.4, SAS Institute, Cary, North Caroline, United State of America).

### 4.4. Ethics Approval and Informed Consent

The study was performed in accordance with the principles of Good Clinical Practice and the ethical standards laid down in the Declaration of Helsinki. The protocol was approved by the Comitato Etico Area Vasta Romagna (n. I5/424 on 15/09/2010) (Protocol code IRST 157.01, Eudract number 2010-020379-22) and written informed consent were obtained from all subjects before they participated in the study.

## 5. Conclusion

In conclusion, findings of favorable OS and disease control, together with an optimal safety profile, suggest that this regimen is a good option in patients with LAPC. The combination of hypofractionated accelerated tomotherapy with potentially more active chemotherapy regimens (i.e., FOLFOXIRI or gemcitabine/albumin-bound paclitaxel) should be investigated further as it might improve survival outcomes further. Our CTRT regimen should also be considered for use in randomized trials.

## Figures and Tables

**Table 1:** Patient characteristics at baseline (No. = 40).

Characteristic	No.	%
Age, years		
Median (range)—67 (41–75)		
Gender		
Male	15	37.5
Female	25	62.5
ECOG Performance Status		
0	25	62.5
1	14	35.0
2	1	2.5
Histological classification		
Adenocarcinoma	35	87.5
Mucinous Adenocarcinoma	2	5
Carcinoma	3	7.5
Stage		
IIA	3	7.5
IIB	8	20.0
III	29	70.0
Tumor site		
Head	25	62.5
Body	13	32.5
Tail	2	5.0
Biliary stent		
No	22	55.0
Yes	18	45.0

**Table 2:** Maximum toxicity in 40 patients treated with chemoradiotherapy (CTRT).

Toxicity	Grade				
	0	1	2	3	4
	No. (%)	No. (%)	No. (%)	No. (%)	No. (%)
Neutropenia	29 (72.5)	6 (15.0)	0	3 (7.5)	2 (5.0)
Febrile neutropenia	39 (97.5)	0	0	0	1 (2.5)
Leucopenia	37 (92.5)	1 (2.5)	2 (5.0)	0	0
Thrombocytopenia	23 (57.5)	2 (5.0)	9 (22.5)	5 (12.5)	1 (2.5)
Anaemia	28 (70.0)	9 (22.5)	1 (2.5)	2 (5.0)	0
Fatigue	17 (42.5)	4 (10.0)	18 (45.0)	1 (2.5)	0
Fever	25 (62.5)	8 (20.0)	3 (7.5)	4 (10.0)	0
Weight loss	37 (92.5)	2 (5.0)	1 (2.5)	0	0
Pain	27 (50.0)	5 (12.5)	14 (35.0)	0	1 (2.5)
Hepatotoxicity	39 (97.5)	1 (2.5)	0	0	0
Peripheral neuropathy	29 (72.5)	4 (10.0)	6 (15.0)	1 (2.5)	0
Allergic reaction	34 (85.0)	2 (5.0)	3 (7.5)	1 (2.5)	0
Nausea	15 (37.5)	5 (12.5)	18 (45.0)	2 (5.0)	0
Vomiting	21 (52.5)	6 (15.0)	12 (30.0)	1 (2.5)	0
Diarrhoea	24 (60.0)	5 (12.5)	8 (20.0)	2 (12.5)	1 (2.5)
Constipation	31 (77.5)	4 (10.0)	5 (12.5)	0	0
Stomatitis	39 (97.5)	1 (2.5)	0	0	0
Alopecia	39 (97.5)	0	0	1 (2.5)	0
Hyporexia	35 (87.5)	4 (10.0)	1 (2.5)	0	0
Dysgeusia	38 (95.0)	1 (2.5)	1 (2.5)	0	0
Rash	36 (90.0)	1 (2.5)	3 (7.5)	0	0

**Table 3:** Clinico-pathological characteristics of resected patients.

Patient	Surgery	Vascular Resection	pTNM	Grading	Vascular or Perineural Invasion	Margin
1	DCP	No	T3N1M0	2	Yes	R0
2	DCP	Yes	T3N1M0	2	Yes	R0
3	TP	No	T1N0M0	2	No	R0
4	DCP	No	T1N0M0	ukn	No	R0
5	DCP	Yes	T1N0M0	2	Yes	R0

Abbreviations. DCP: Duodenocefalopancreasectomy; TP: Total Pancreasectomy; R0: R0- no cancer cells seen microscopically at the resection margin; ukn: unknown.
