# Erratum: Passardi, A. et al. Chemoradiotherapy (Gemox Plus Helical Tomotherapy) for Unresectable Locally Advanced Pancreatic Cancer: A Phase II Study. *Cancers* 2019, *11*, 663

**DOI:** 10.3390/cancers12010178

**Published:** 2020-01-10

**Authors:** Alessandro Passardi, Emanuela Scarpi, Elisa Neri, Elisabetta Parisi, Giulia Ghigi, Giorgio Ercolani, Andrea Gardini, Giuliano La Barba, Flavia Pagan, Andrea Casadei-Gardini, Martina Valgiusti, Fabio Ferroni, Giovanni Luca Frassineti, Antonino Romeo

**Affiliations:** 1 Department of Medical Oncology, Istituto Scientifico Romagnolo per lo Studio e la Cura dei Tumori (IRST) IRCCS, Via P. Maroncelli n. 40, 47014 Meldola, Italy; alessandro.passardi@irst.emr.it (A.P.); casadeigardini@gmail.com (A.C.-G.);; 2 Unit of Biostatistics and Clinical Trials, Istituto Scientifico Romagnolo per lo Studio e la Cura dei Tumori (IRST) IRCCS, Via P. Maroncelli n. 40, 47014 Meldola, Italy; flavia.pagan@irst.emr.it; 3 Radiotherapy Unit, Istituto Scientifico Romagnolo per lo Studio e la Cura dei Tumori (IRST) IRCCS, Via P. Maroncelli n. 40, 47014 Meldola, Italy; elisa.neri@irst.emr.it (E.N.); elisabetta.parisi@irst.emt.it (E.P.); giulia.ghigi@irst.emr.it (G.G.);; 4 General and Oncologic Surgery Unit, Morgagni-Pierantoni Hospital, AUSL Romagna, Via C. Forlanini n. 34, 47121 Forlì, Italy; giorgio.ercolani@auslromagna.it (G.E.); andrea.gardini@auslromagna.it (A.G.); giuliano.labarba@tin.it (G.L.B.); 5 Department of Medical and Surgical Sciences, University of Bologna, Via Massarenti n. 9, 40138 Bologna, Italy; 6 Radiology Unit, Istituto Scientifico Romagnolo per lo Studio e la Cura dei Tumori (IRST) IRCCS, Via P. Maroncelli n. 40, 47014 Meldola, Italy

The authors wish to make the following corrections to this paper [1]:

The institute name in affiliations 2, 3 and 6 in [1] “IRST-IRCCS” should be written in full, and changed to “Istituto Scientifico Romagnolo per lo Studio e la Cura dei Tumori (IRST) IRCCS”.

The authors would like to apologize for any inconvenience caused to the readers by these changes.
